# Quercetin mitigates rheumatoid arthritis by inhibiting adenosine deaminase in rats

**DOI:** 10.1186/s10020-022-00432-5

**Published:** 2022-02-22

**Authors:** Karim Samy El-Said, Amira Atta, Maysa A. Mobasher, Mousa O. Germoush, Tarek M. Mohamed, Maha M. Salem

**Affiliations:** 1 Biochemistry Division, Chemistry Department, Faculty of Science, Tanta University, Tanta, 31527 Egypt; 2 Pathology Department, Biochemistry Division, College of Medicine, Jouf University, Sakaka, 41412 Saudi Arabia; 3 Biology Department, College of Science, Jouf University, Sakaka, 41412 Saudi Arabia

**Keywords:** Adenosine deaminase, Rheumatoid arthritis, Quercetin, Fenugreek, Methotrexate

## Abstract

Rheumatoid arthritis (RA) is a chronic inflammatory joint disease characterized by synovial proliferation and bone destruction. Adenosine deaminase (ADA) is a key inflammatory enzyme that increases joint stiffness and pain in RA. In this study, we evaluated the in-silico, and in vivo inhibitory effect of quercetin isolated from Egyptian Fenugreek on ADA enzyme activity. We also determined the combinatorial effect of quercetin on methotrexate mediated anti-inflammatory efficacy and toxicity. In-silico molecular docking was conducted and confirmed in an in vivo RA rat model. The results showed that the inhibition constant of quercetin on joint ADA by docking and in-vitro was 61.9 and 55.5 mM, respectively. Therefore, quercetin exhibits anti-inflammatory effect in a rat RA model as evidenced by reducing the specific activity of ADA in joint tissues, lower jaw volume, enhance body weight, downregulate ADA gene expression, reduce levels of RA cytokines interleukin-1^β^, interleukin-6, tumor necrosis factor-α, also, rheumatoid factor, C-reactive protein, and anti-cyclic citrullinated peptide RA biomarker levels. These findings demonstrate that the purified quercetin has a promising anti-inflammatory effect against RA disease through its inhibitory effects on the ADA enzyme. Furthermore, isolated quercetin improved the anti-inflammatory efficacy of methotrexate, reduced its toxic effects by increasing antioxidant enzymes and reducing oxidative stress.

## Introduction

Rheumatoid arthritis (RA) is a chronic autoimmune condition caused by combination of hereditary, epigenetic, nongenetic, and environmental factors (McInnes and Schett 2017). RA attacks cartilage and bone, causing joint damage and dysfunction (McInnes and Schett 2017). Overactivation of B and T lymphocytes, macrophages, synovial-like fibroblasts, matrix metalloproteinase (MMP) release and the production of interleukin-1^β^ (IL-1^β^), interleukin-6 (IL-6), and tumor necrosis factor α (TNF-α) cytokines results in pain, atrophy, deformation of joints, bones erosion, and osteoporosis. RA-induced dysfunction and disability, may even lead to premature death over the long term (Scott and Kingsley 2006).

Adenosine deaminase (ADA) has been considered as a biomarker for the inflammatory process in RA patients (Zamani et al. 2012; Nalesnik et al. 2011). ADA represents a checkpoint for the control of the immune system through the modulation of adenosine pathways (Nalesnik et al. 2011). Moreover, the ADA enzyme plays an important role in the maturation and differentiation of the lymphoid system. Previous study has described the functional implications of ADA as well as the design of several ADA inhibitors (Cristalli et al. 2001).

Methotrexate (MTX) is considered as the standard treatment for RA because of its anti-inflammatory and immunosuppressive properties (Bedoui et al. 2019). However, it has many limitations as it negatively affects the normal cells resulting in several toxicities. Also, the adverse effects of MTX on the neuronal, gastrointestinal, reproductive, respiratory, urinary, cardiovascular, and immune systems have been reported (Cronstein and Aune 2020; Salem et al. 2020). Therefore, identifying an effective natural anti-inflammatory agent for RA treatments without side effects is an active area of investigation.

Quercetin is a flavonoid found in fruits and vegetables and is known for its anti-inflammatory and antioxidant effects (Haleagrahara et al. 2009; Mahmoud et al. 2013). Quercetin suppresses the clinical symptoms of arthritis by inhibiting the release of inflammatory cytokines, decreasing lipopolysaccharide-induced cyclooxygenase (COX-2) levels, and antagonizing and bone resorption by suppressing of nuclear factor-kappa β (NF-kβ) and AP-1 activity (Kim et al. 2019). It prevents the recruitment of macrophages and neutrophils, as well as synoviocyte proliferation (Gardi et al. 2015; Haleagrahara et al. 2018). Based on these findings, the present study evaluated the impact of quercetin on ameliorating the synovial inflammation by inhibiting ADA enzyme activity in a rat model of RA. Furthermore, the combinatorial effect of quercetin on MTX anti-inflammatory and toxicity in the RA model was evaluated.

## Materials and methods

### Materials

Aluminum chloride, potassium acetate, lead acetate, adenosine, phenol, sodium nitroprusside, Coomassie brilliant blue, silica gel (60–120 mesh), quercetin, Sephadex (G-100) and complete Freund’s adjuvant (CFA) were purchased from Sigma-Aldrich. Methotrexate (25 mg/ml) was purchased from EIMC United Pharmaceutics, Cairo, Egypt. Fenugreek seeds (Giza 1, Egyptian cultivar) were purchased from a local market of Tanta, Egypt.

### In-silico study

The three-dimensional *Mus musculus* ADA enzyme structure was obtained from a protein data bank (PDB ID: 1a4m). The energy of the ADA protein structure was minimized by applying an OPLS-3 force field (Harder et al. 2016). The chemical structure of quercetin was obtained from the PubChem database and prepared using the Schrodinger program (Halgren and Nachbar 1996). The enzyme-ligand interaction study was performed using Molegro Virtual Docker (2008). The Discovery studio 3.5 tool was used to visualize the intermolecular interactions between the ADA enzyme and quercetin.

### Extraction and identification of quercetin from fenugreek ethanolic extract

Fenugreek seeds were dried, ground and 50 g were extracted with 500 ml of 70% ethanol. The collected extracts were filtered and the aqueous ethanolic extract was concentrated using a rotary evaporator at 40 °C and stored at − 20 °C until further use (Dua et al. 2013). The quercetin was isolated by adding 10 g of Fenugreek ethanolic extract to a silica gel column (60–120 mesh). The elution was initiated with 100% hexane and then the polarity was increased with hexane, ethyl acetate and ethanol at ratios of 80:20, 75:25 and 50:50, respectively. The eluted fractions were collected and dried to obtain a yellow amorphous powder. The isolated quercetin was then identified using thin layer chromatography and its RF value was compared with that of standard quercetin. In addition, UV Spectra were obtained at 257 nm, 307 nm, and 432 nm. Moreover, using Perkin Elmer spectrophotometer, the KBr technique was performed to check the Fourier transform infrared (FT-IR) spectra to confirm the structure of the isolated quercetin compared with the standard.

### Determination of ADA enzyme activity and protein concentration in rat joints

The crude ADA enzyme was extracted by homogenizing 200 mg of joint tissue in 1.5 ml of 50 mM potassium phosphate buffer, pH 7.5, containing 150 mM sodium chloride using a Teflon pestle homogenizer at 4 °C. The homogenate was centrifuged at 8000×*g* for 15 min and sonicated for 15 min at 4 °C. The crude ADA enzyme supernatant was collected, and its enzymatic activity was estimated. The ADA activity was determined by the Guisti (1974) method with modifications (Guisti 1974). The reaction mixture contained 21 mM adenosine substrate solution, 50 mM potassium phosphate buffer, pH 7.5 and joint homogenate (10%). The enzyme reaction was incubated at 37 °C for 15 min., stopped with the addition of phenol/sodium nitroprusside solution (106 mM phenol; 0.17 mM sodium nitroprusside) and alkaline hypo-chloride solution (11 mM NaOCl; 125 mM NaOH) then incubate at 37 °C for 30 min. The absorption was measured at 628 nm. The protein content was measured by the Bradford method (Bradford 1976).

### Purification of ADA enzyme from rat joint tissue

The crude ADA enzyme was precipitated using 20% ammonium sulfate saturation, centrifuged at 10,000×*g* for 30 min. at 4 °C. The precipitate was dissolved in 50 mM potassium phosphate buffer, pH 7.5, then dialyzed overnight. The dialysate was subjected to Sephadex G-100 gel filtration column chromatography (2 × 87 cm). Fractions were collected at a flow rate of 3 ml/5 min. For each fraction, the total protein content was measured at 280 nm. The fractions containing high protein content were pooled and assayed for ADA enzyme activity. Finally, the fractions enriched with ADA activity were pooled for kinetics measurements (Mohamed 2006; Abu-Khudir et al. 2019).

### Kinetic inhibition of ADA enzyme by quercetin in rat joint

Quercetin at various concentrations (0.1–1 mM) was added to the partially purified ADA enzyme, incubated for 1 h then its activity was measured as described in section ‘In vivo studies’. The concentrations of quercetin that inhibited ADA by 40%, 50% and 60% were selected and added to various concentrations (5–150 mM) of adenosine and the enzyme activity was measured. The Km, Vmax and Ki kinetic measurements were according to Mohamed (2006).

### In vivo studies

#### Rats

Seventy adult Wistar male rats (150–170 g) were obtained from the Faculty of Agriculture, Alexandria University (Alexandria, Egypt). The animals were housed under a 12:12 h light–dark cycle at a controlled temperature of 20–22 °C and maintained for a 1-week acclimatization period with a pellet diet and drinking water ad libitum. Our study was carried out according to the guidelines approved by the Research Ethical Committee (Faculty of Science, Tanta University, Egypt) (IACUC-SCI-TU-0161).

#### Experimental design

Seventy adult Wistar male rats were classified into seven groups (n = 10) as follows: Gp1 (Normal control): Rats were subcutaneously injected with 1.5 ml saline into the sub-plantar region of the right hind foot paw; Gp2 (Rheumatoid Arthritis): Rats were injected with a single subcutaneous injection of 0.1 ml complete Freund’s adjuvant (CFA) into the sub-plantar region of the right hind foot paw to induce arthritis, inflammation began to appear shortly after the injection and peaked after 7 days (Sindhu et al. 2012). Gp3 (Methotrexate control): Rats were injected intra-peritoneal (i.p) with 100 ml of 0.75 mg/kg MTX twice—per week for 3 weeks (Anderson et al. 1994). Gp4 (Quercetin control): Rats were injected i.p. with 200 µl of 100 mg/kg quercetin three times—per week for 3 weeks (Firdous 2014). Gp5 (Methotrexate treatment): Rats were injected with CFA as in Gp2 then injected i.p. with 100 µl of 0.75 mg/kg MTX twice—a week for 3 weeks. Gp6 (Quercetin treatment): Rats were injected with CFA as in Gp2 then injected i.p. with 200 µl of 100 mg/kg quercetin three times—per week for 3 weeks. Gp7 (Combination treatment): Rats were injected with a with CFA as in Gp2 then injected with MTX twice—a week and quercetin three times—per week for 3 weeks.

Rats were re-examined every 4 days for paw volume and articular score measurements for the injected paw. At the end of the experiment (28 days after the immunization), the rats were sacrificed by cervical decapitation and blood samples were collected for hematological studies. Sera were separated for biochemical assays and foot paw tissues were immediately removed and a portion was added to 10% buffered formalin for histopathological studies. The other part of the foot paw tissue was homogenized and frozen at − 20 °C for further analyses.

#### Determination of paw thickness, body weight changes and live animal imaging X-ray

The paw thickness was measured according to Zuo et al. (2014) using a digital Vernier caliper on days 7–28 and expressed in mm. Changes in body weight were estimated on days 1–28. On the 28th day, rats were anesthetized with 10% chloral hydrate (0.3 ml/100 mg, i.p.) and X-ray photos were taken of the ankle joint and paws in the Bruker imaging station (Bruker in vivo Multispectral FX PRO, USA).

#### Biochemical analysis

Serum c-reactive protein (CRP), serum Rheumatoid Factor (RF), serum TNF-α, serum IL-6, serum IL-1^β^, serum anti-cyclic citrullinated peptide antibody (anti-CCP) as markers of acute inflammatory phase proteins were measured using sandwich ELISA kits and serum ADA activity was measured using competitive ELISA kits (Sigma Chemical Co., USA) according to the manufacturer’s instructions. ADA enzyme activity was estimated in joint tissue homogenate as described in section ‘In vivo studies’. Malondialdehyde (MDA) levels and glutathione peroxidase (GPx) activity were measured in joint tissue homogenates using Bio-diagnostic kits according to the manufacturer’s instructions.

#### Real-time polymerase chain reaction (RT-PCR) analysis

Quantitative PCR was done as previously described by Kvastad et al. (2015). The specific primer sequences for the ADA gene were GTCCTCTGATTGGATGTCTTGG (forward) and CCAGTCACCAGCTGCTTTAT (reverse). The TGTGTCCGTCGTGGATCTGA forward and CCTGCTTCACCACCTTCTTGA reverse primers were used to amplify GAPDH. Real-time PCR was performed using Power SYBR Master Mix (Thermo Fisher Scientific, US) on an Applied Biosystems 7500 system (Foster City, US). All data were then normalized to GAPDH housekeeping gene. Critical threshold (Ct) values for target gene were normalized to that of GAPDH. The fold change in gene expression was calculated as described previously (Livak and Schmittgen 2001; Noser et al. 2021).

#### Histopathological investigation

Ankle rat joints were added to 10% buffered formalin for 24 h and subsequently, decalcified in 5% formic acid. Then, joint tissues were stained with hematoxylin–eosin, and observed under light microscope to determine the presence of hyperplasia of the synovium, inflammatory cells, and major damage to the joint space.

### Statistical analysis

The experimental data were expressed as the mean ± SE. The significance of differences among the various treated groups and control were analyzed using one-way ANOVA followed by Tukey’s test using GraphPad Prism software 6 (San Diego, CA), and *p*-value < 0.05 were considered statistically significant.

## Results

### In-silico molecular docking study

**Fig. 1:**
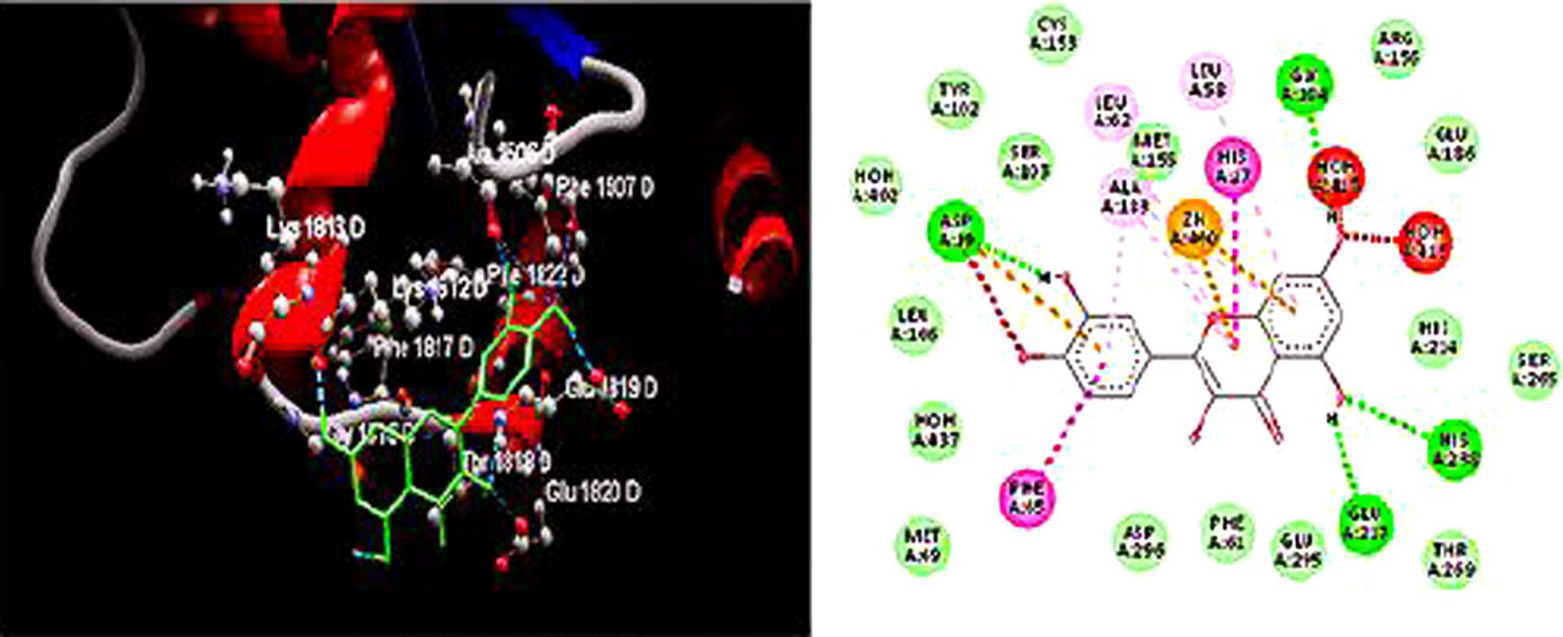
The interaction between quercetin and the active site of adenosine deaminase. The left side represents 3D and the right side represents the 2D complex enzyme ligand interaction

The optimal molecular structure of quercetin as inhibitor of adenosine deaminase enzyme was determined. The active sites of ADA enzyme were cleaned up from any native ligand present, then quercetin was docked into the binding site of ADA enzyme. The ADA and quercetin binding dimensions were x = 54.80 Å, y = 45.06 Å and z = 77.09 Å, with a 9 Å grid spacing. Docking calculations of quercetin showed 4H-bond interactions with Lys1812, Glu1820, Ala1506, and Glu1819 as important amino acid residues and H-bond distances of 2.86, 3.29, 2.87 and 3.0047 Å, respectively, (Fig. 1). This theoretically explains the inhibitory effect of quercetin on ADA enzyme. The minimum energy of quercetin docked to the ADA enzyme active site was observed from the molecular model (Table 1).

**Table 1:** The calculated molecular docking parameters

Parameter	BE	Ki	MDS	RS	HB
Inhibitor	− 95.929	61.9	− 98.715	− 74.444	− 11.929

*BE* binding interaction, *Ki* inhibition constant, *MDS* molecular docking score, *RS* Rerank score, *HB* hydrogen bond interaction energy

### Isolation and characterization of quercetin from Fenugreek extract

**Fig. 2:**
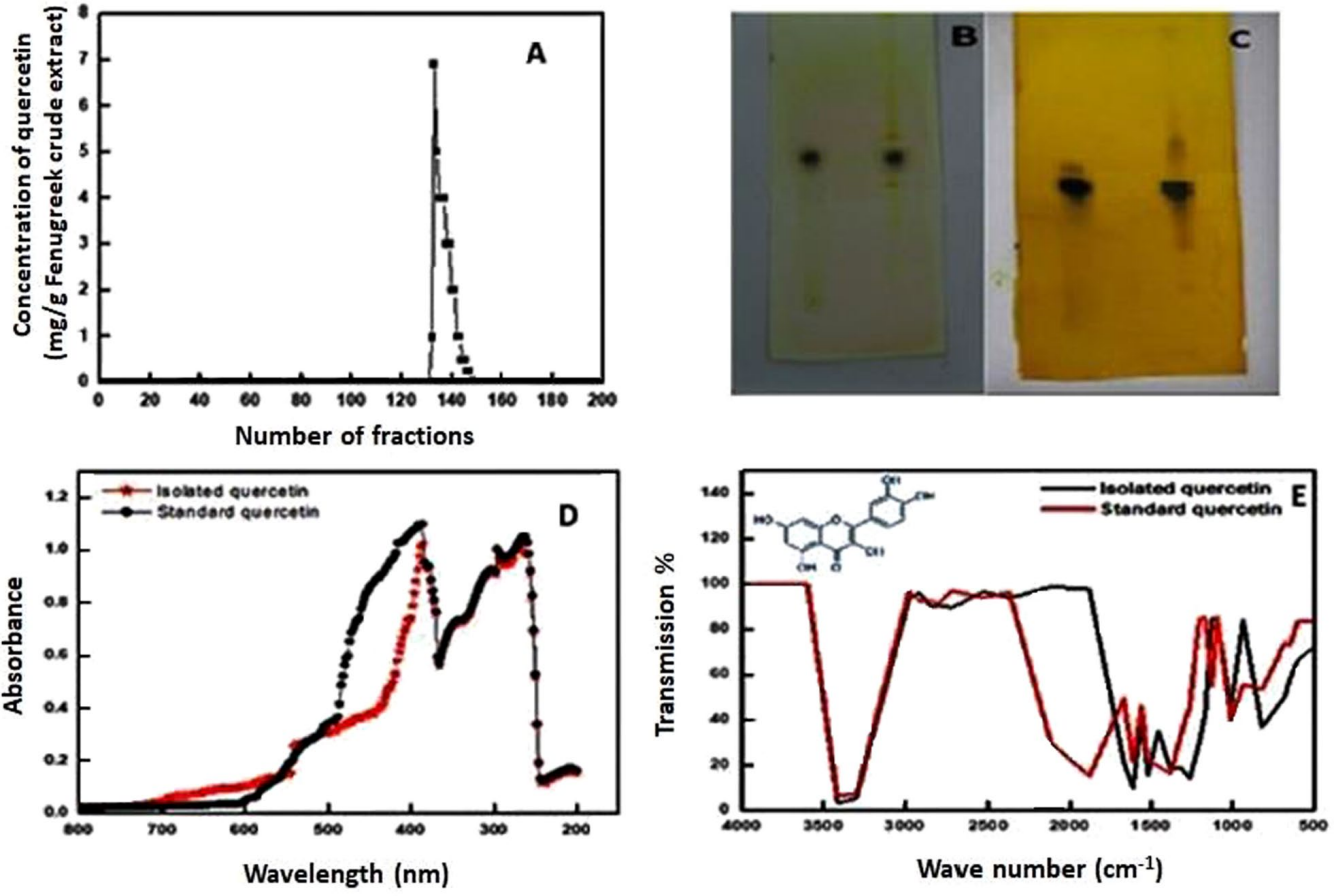
**A** Isolation of quercetin from fenugreek extract using a silica gel column, **B** TLC of the isolated and standard quercetin using iodine spray, **C** TLC of isolated and standard quercetin using FeCl_3_ spray, **D** UV–vis spectroscopy, and **E** FTIR spectrum

Quercetin was isolated from Fenugreek extract using silica gel column chromatography. It appeared in fractions of hexane/ethyl acetate/ethanol as shown in Fig. 2A. Furthermore, the isolated fractions of quercetin and standard quercetin showed the presence of same brown spot with an R_f_ value of 0.52 after the plate was sprayed with iodine (Fig. 2B) and 5% FeCl_3_ (Fig. 2C). Moreover, UV–vis spectroscopy initially confirmed the isolation of quercetin from Fenugreek extract with peaks at 386, 296 and 262 nm compared with standard quercetin (Fig. 2D). Also, the isolated quercetin was analyzed by FTIR which revealed the presence of a C–O–C bond at 1009 cm^−1^, C=C bond at 1610 cm^−1^, C=O bond at 1667 cm^−1^ and an O–H bond at 3406 cm^−1^ when compared with standard quercetin (Fig. 2E).

### ADA purification from rat joints

**Fig. 3:**
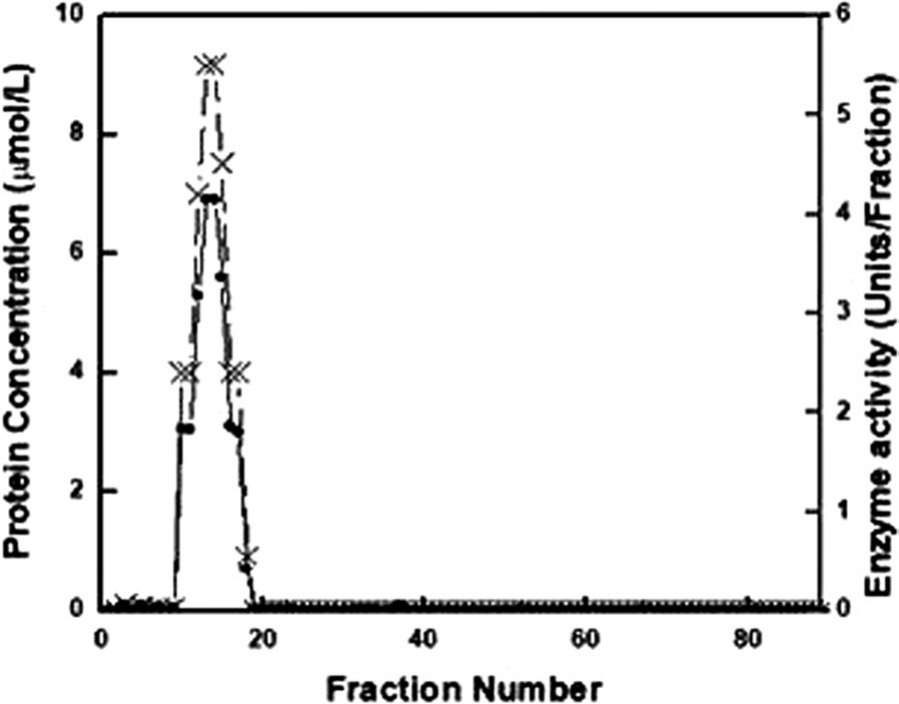
Elution profile of ADA from rat joints (gel filtration chromatography on Sephadex G-100 HR)

The partially purified ADA enzyme was determined to have a normal value in the rat joint homogenate. The crude ADA enzyme was precipitated with 20% ammonium sulfate, dialyzed, then applied to Sephadex G-100 High Resolution (HR) column. As illustrated in Table 2, the specific activity of ADA increased from 0.0058 to 5.913 U/mg with 996-fold and 13% recovery. The elution profile from the Sephadex G-100 HR column is shown in Fig. 3. Kinetic measurements were carried out on the ADA enzyme resulting from Sephadex column based on the highest fold purification.

**Table 2:** Partial purification of ADA from rat joint tissues

Steps	Volume (ml)	Total protein (mg)	Total activity (mU)	Specific activity (mU/mg)	Purification fold	Recovery %
Crude homogenate	10	16,080	94.3	0.0058	1	100
High speed supernatant	9.3	2848	43.65	0.015	2.58	46.2
20% Ammonium sulfate	4.2	737.26	17.947	0.025	4.31	18.98
Dialysis	3.2	18.42	14.95	0.81	137.28	15.85
Gel filtration Sephadex (G-100)	8.2	2.08	12.3	5.913	996	13

### Kinetic inhibition of joint's ADA by quercetin

**Fig. 4:**
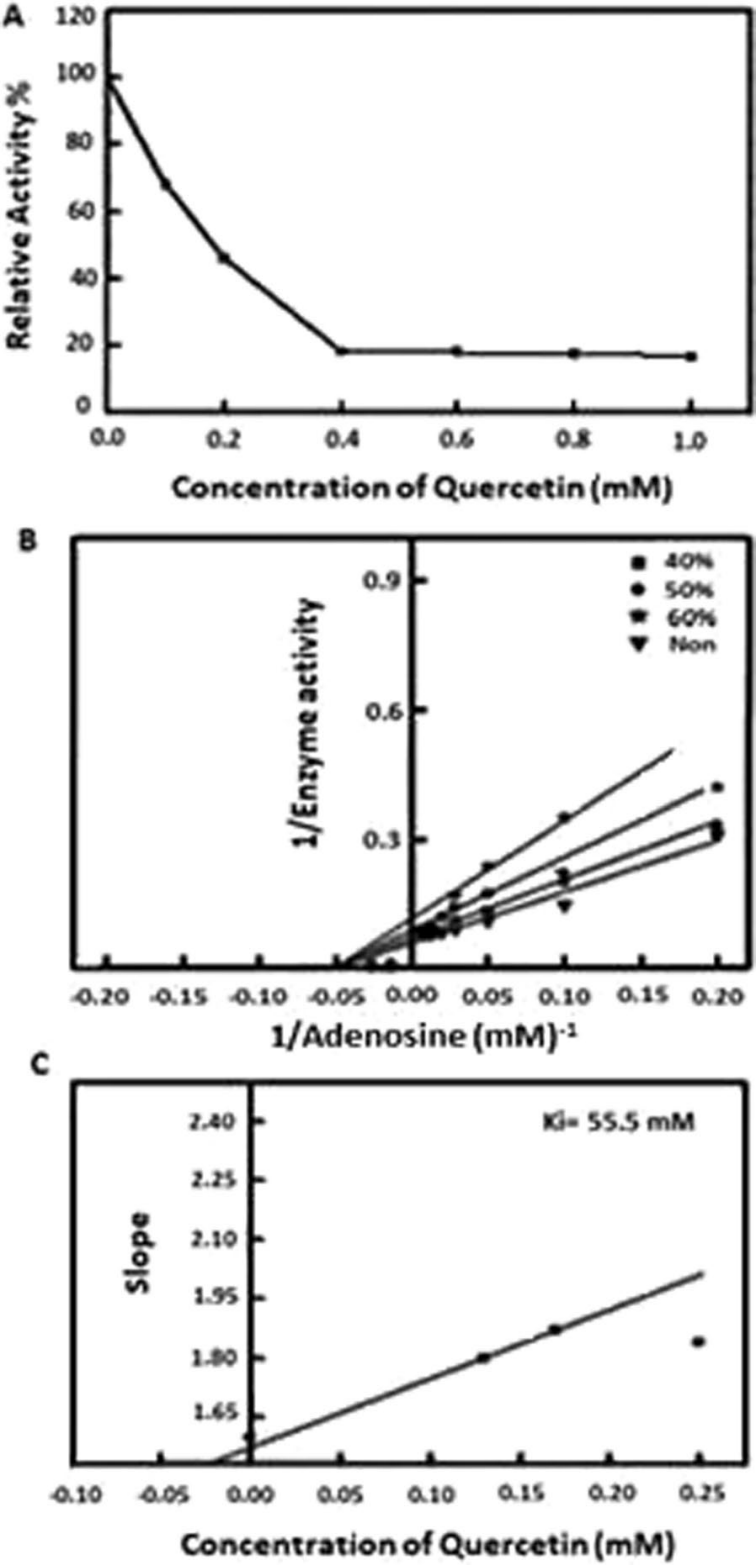
**A** Inhibition of ADA enzyme activity by various concentration of quercetin. **B**, **C** Lineweaver Burk blot and the inhibition constant (Ki) of ADA by quercetin

The ADA enzyme was inhibited by quercetin with 50% inhibition at concentration of 0.17 mM as shown in Fig. 4A. Following the Lineweaver Burk plot, quercetin at concentrations 0, 0.25, 0.17, and 0.13 mM exhibited inhibition as the V_max_ increased whereas K_m_ was fixed as shown in Fig. 4B. Therefore, the inhibition type was non-competitive. The K_i_ value was 55.5 mM, this value was like the results of the in-silico docking study in which a value of 61.6 mM was observed (Fig. 4C).

### In vivo study

#### Change in paw volume

The results showed that there was no effect on paw volume (PV) in all control and treated groups at day 0 of administration. On day 7, PV was significantly (*p* < 0.0001) increased in the untreated RA control group and those treated with either MTX, QUE alone or combined groups compared with the normal, MTX and QUE control groups. Following treatment on day 14, 21 and 28 there was a marked (*p* < 0.0001) reduction in PV in MTX and QUE alone treated groups with a minimal reduction in the combined group compared with the untreated RA control group (Table 3).

**Table 3:** Effect of administration of QUE and/or MTX on paw volume

Groups	Day (0)	Day (7)	Day (14)	Day (21)	Day (28)
Normal control	0.11 ± 0.009	0.133 ± 0.0045	0.137 ± 0.004	0.1331 ± 0.005	0.129 ± 0.003
Rheumatoid arthritis control	0.115 ± 0.004	1.032 ± 0.0078****	0.809 ± 0.013****	0.863 ± 0.024****	0.809 ± 0.013****
Methotrexate control	0.116 ± 0.006	0.13 ± 0.0058	0.102 ± 0.005	0.073 ± 0.002	0.070 ± 0.008
Quercetin control	0.122 ± 0.002	0.135 ± 0.0042	0.152 ± 0.002	0.132 ± 0.001	0.131 ± 0.001
Methotrexate treatment	0.127 ± 0.002	1.031 ± 0.0137****	0.898 ± 0.052****	0.408 ± 0.030^+++,^****	0.291 ± 0.029^++++,^**
Quercetin treatment	0.122 ± 0.002	1.026 ± 0.0096****	0.72 ± 0.032****	0.51 ± 0.048^++,^****	0.44 ± 0.030^+++,^***
Combination treatment	0.128 ± 0.002	1.032 ± 0.0079****	0.869 ± 0.016****	0.369 ± 0.016^++++,^***	0.233 ± 0.030^++++,^**

*p* < 0.05 is considered significant, where ^+^: significantly different from the RA control group and *: significantly different from the normal control groups. Results are expressed as mean ± SE, (n = 8)

#### Body weight changes

**Fig. 5:**
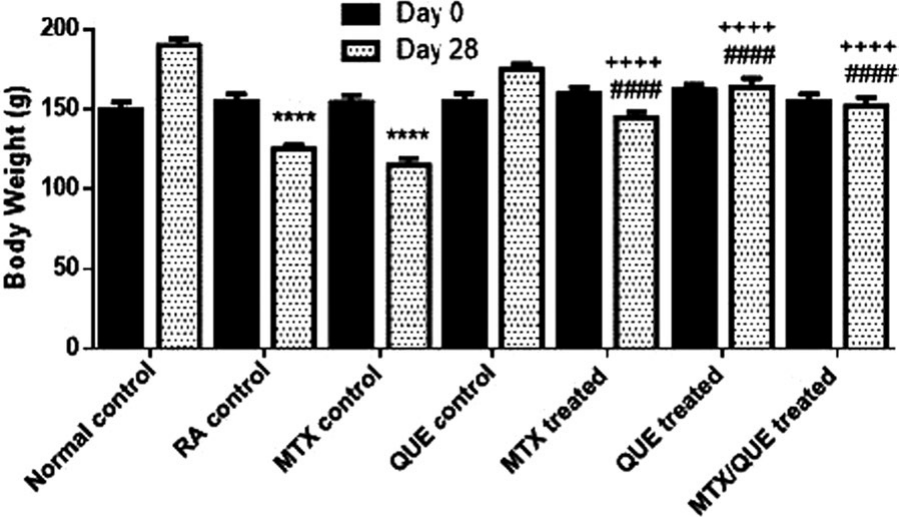
The body weight of rats at the start and at the end of the experiment after treatment with QUE, MTX or their combination. Values are expressed as the mean ± SE (n = 8) of experimental group. *p < 0.05 showed significance vs. normal control, ^+^*p* < 0.05 showed significance vs. the RA control group, ^#^*p* < 0.05 showed significance vs. the MTX control group

Body weight at day 0 was almost identical among all the treated and control groups, whereas it was significantly (*p* < 0.0001) diminished in the untreated RA and MTX control groups compared with the normal control group during the subsequent period. Following treatment, the body weight at day 28 was significantly (*p* < 0.0001) enhanced in either MTX, QUE alone treated group or in the combined group compared with the untreated RA and MTX control groups (Fig. 5).

#### Live animal imaging and X-ray

**Fig. 6:**
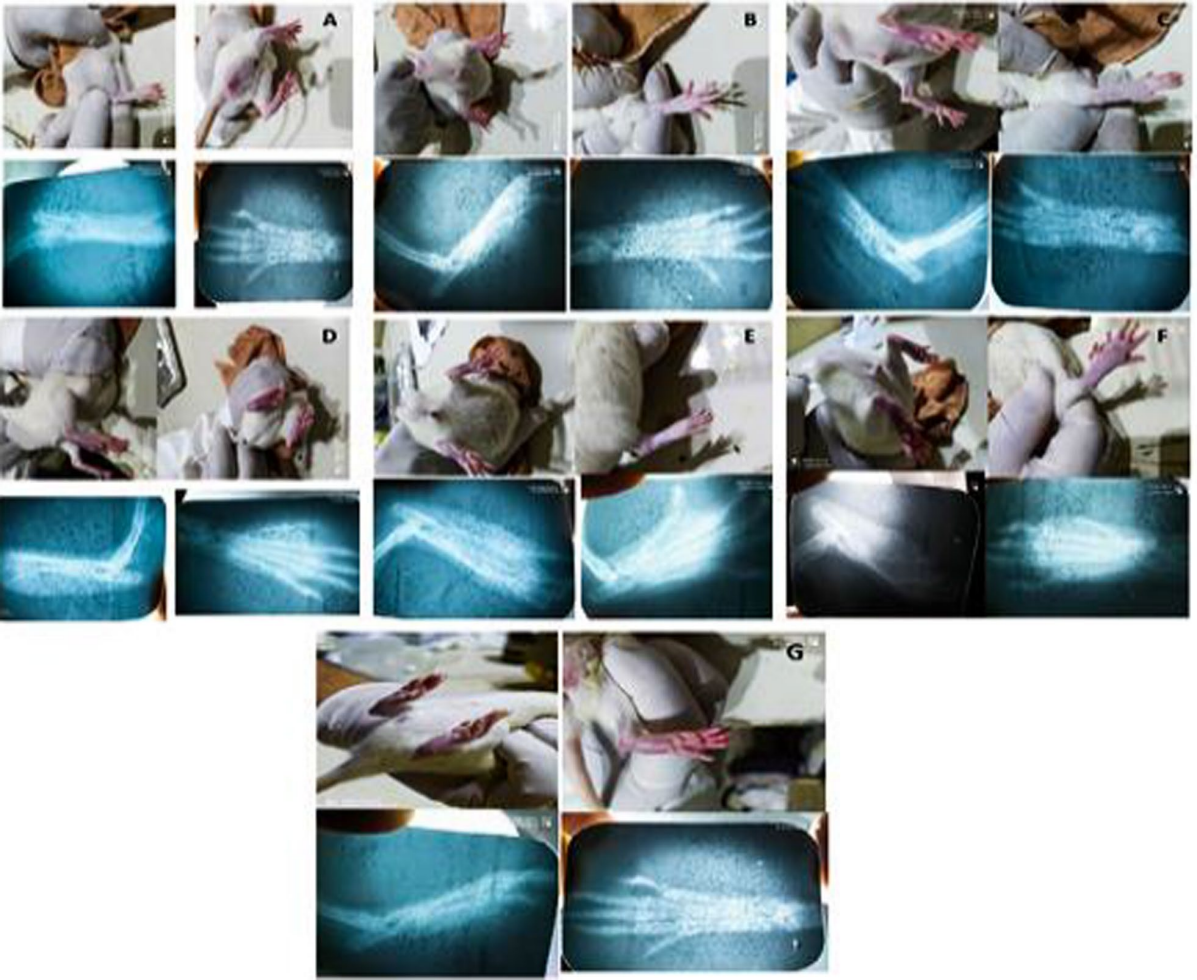
Live animal imaging and X-ray **A** normal control group, **B** RA control group, **C** MTX control group, **D** QUE control group, **E** MTX treated group, **F** QUE treated group, **G** MTX/QUE combinatorial treated group

Our results showed that, the MTX and QUE control groups exhibited no significant change in joint tissue compared with normal control group. While the RA-induced joint showed narrowing of the joint space and, soft swelling tissue compared with the normal control group. Moreover, the soft swelling tissue was diminished and, the joint space was intact in rats treated with either MTX, QUE alone or by combinatorial treatment compared with untreated RA control group (Fig. 6).

#### RA sera analysis

**Fig. 7:**
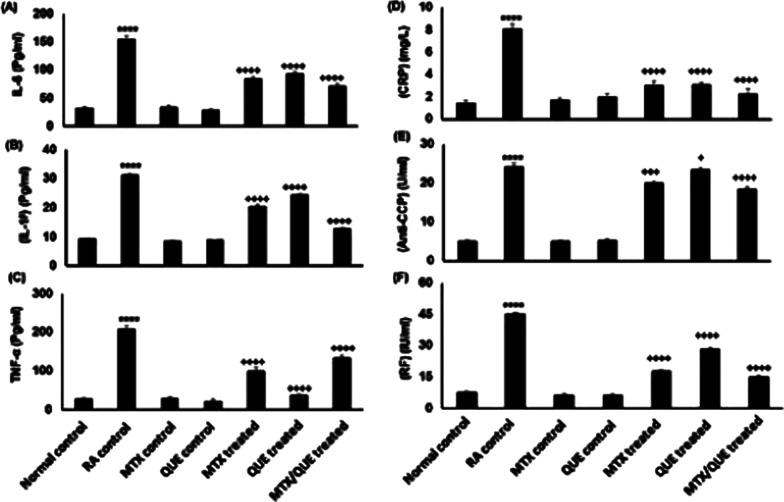
Levels of Il-6, 1β, TNF-α, CRP, CCP, and RF in the sera of different groups. Data are expressed as the means ± S.E. *p < 0.05 showed significance vs. the normal control, ^+^*p* < 0.05 showed significance vs. the RA group

The sera levels of interleukin 6, 1^β^, tumor necrosis factor alpha (TNF-α), C-reactive protein (CRP), anti-CCP, rheumatoid factor (RF) were significantly (*p* < 0.0001) increased in the untreated RA control group as compared with the normal control group. These sera levels were significantly (*p* < 0.0001) decreased in all treated groups with minimal dimmish in the combined group compared with the untreated RA control group (Fig. 7).

#### Quercetin improves the antioxidant capacity in rat joint tissues

**Fig. 8:**
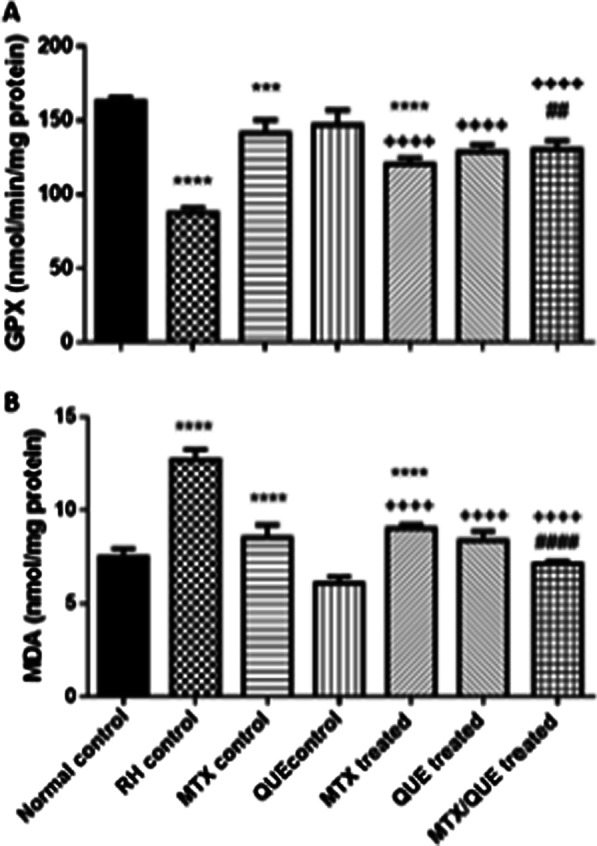
The levels of GPx (**A**) and MDA (**B**) post treatment with QUE or MTX, or their combination. Values are expressed as the mean ± SE. *p < 0.05 showed significance vs. the normal control, ^+^*p* < 0.05 showed significance vs. the RA control group, ^#^*p* < 0.05 showed significance vs. the MTX treated group

Glutathione peroxidase (GPx) activity was increased significantly in QUE-treated group (*p* < 0.0001) and in combined treated group (*p* < 0.001) compared with the untreated RA control and MTX alone treated group. GPx activity was significantly decreased in untreated RA control group (*p* < 0.0001) and the MTX alone control and treated groups (*p* < 0.001) compared with the normal control group. Malonaldehyde (MDA) levels were remarkably reduced in rats treated either with QUE (*p* < 0.0001) or its combination (*p* < 0.0001) compared with the RA untreated control group and MTX alone treated group. This lipid peroxidation marker was increased in untreated RA control group (*p* < 0.0001) and MTX alone control and treated groups (*p* < 0.0001) compared with the normal control group (Fig. 8).

#### Quercetin inhibits the ADA enzyme alone or in combination with MTX

**Fig. 9:**
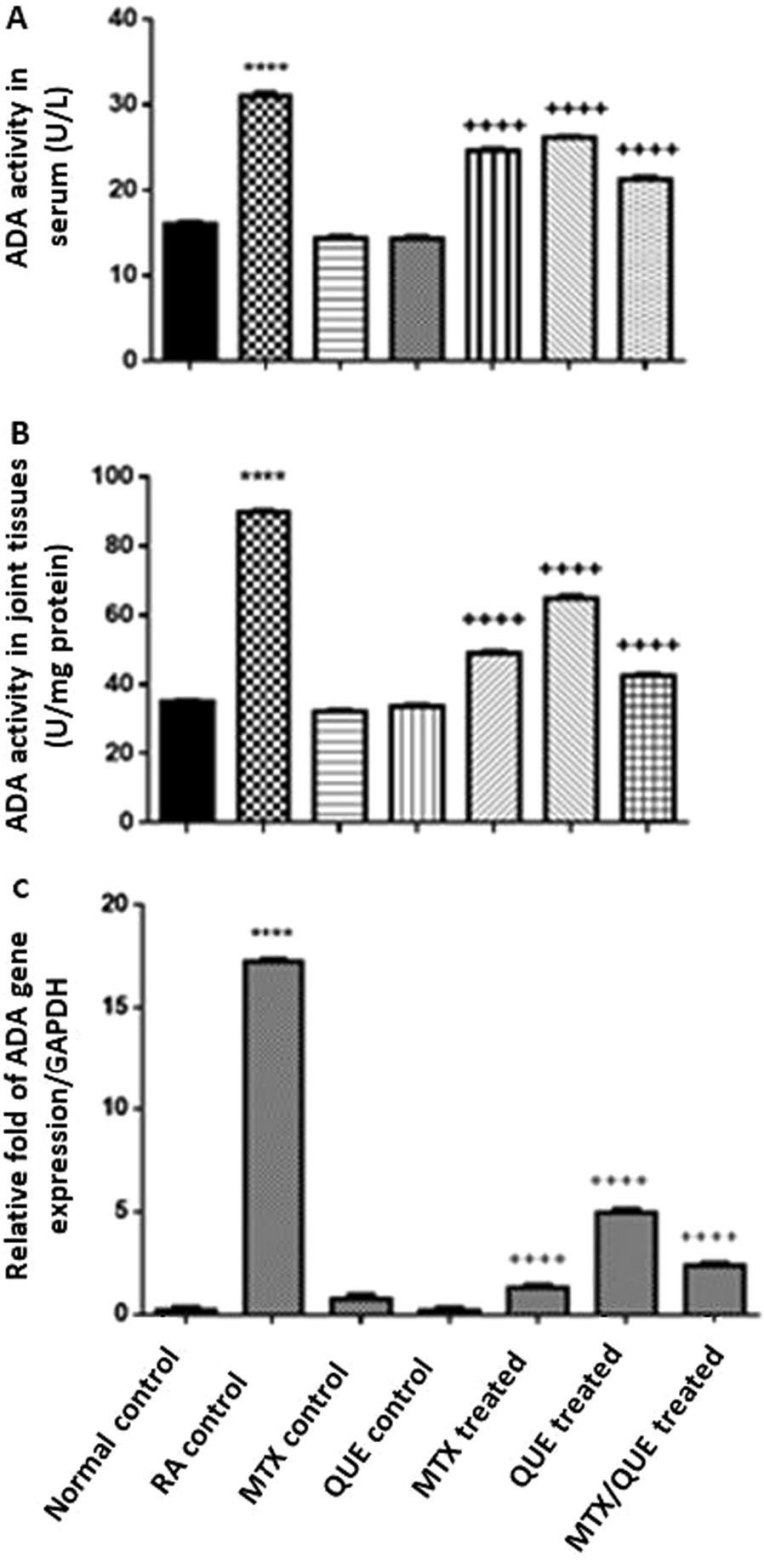
**A** ADA enzyme activity in rat’s sera. **B** ADA enzyme activity in rat's joints tissues, **C** Relative expression of ADA in rat joint tissues. Data are expressed as the means ± S.E. (n = 8) *p < 0.05 showed significance vs. the normal control, ^+^*p* < 0.05 showed significance vs. the RA control group

Our results illustrated that, ADA enzyme activity and gene expression were remarkably (*p* < 0.0001) increased in rat sera and joints of the untreated RA control group because of the swelling and inflammation occurred compared with the normal control group. There was no significant change observed in the sera and joints of the MTX and QUE alone control groups compared with the normal control group. In contrast, ADA enzyme activity and gene expression were significantly (*p* < 0.0001) inhibited in rat sera and joints treated with the QUE or MTX alone or in combination with minimal inhibition in the combined group compared with the untreated RA control group. These results were consistent with the in silico and kinetic studies (Fig. 9).

#### Histological assessment of ankle joints

**Fig. 10:**
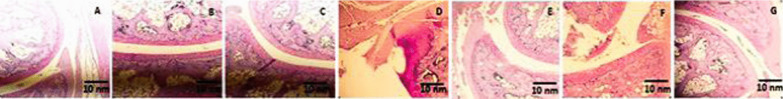
Histopathological assessment of the **A** normal control group, **B** RA control group, **C** MTX control group, **D** QUE control group, **E** MTX treated group, **F** QUE treated group, **G** MTX/QUE combinatorial treated group

Histopathological analysis exhibited severe signs of arthritis associated with bone erosion, synovial hyperplasia, inflammatory cell infiltration, and joint fusion of the untreated RA control ankle joint tissue group compared with normal control group. Also, the treated QUE and MTX either alone or in combination were able to recover from these effects compared with the untreated RA control group (Fig. 10).

## Discussion

Quercetin (QUE) inhibits the oxidation of biomolecules and activates or inhibits several proteins. For instance, QUE inhibits the non-specific protein kinase enzyme (Williams et al. 2004). QUE has also been shown to act as an agonist of the G protein-coupled receptor (Prossnitz and Barton 2014). Previous studies have demonstrated that QUE is a potential agent for the treatment of RA, however, the underlying antiarthritic mechanism of QUE has not been fully elucidated. Therefore, the present study addressed the molecular and biochemical mechanisms of QUE alone or in combination with MTX on the ADA enzyme in an arthritic rat model. According to the in-silico study, QUE showed favorable binding with the ADA enzyme. QUE was isolated from Fenugreek seeds extract using silica gel column chromatography and it was resolved into fractions containing hexane/ethyl acetate/ethanol. This was done in accordance with Sambandam et al. (2016) who isolated QUE from the leaves of *Trigonella foenum-graecum* (Sambandam et al. 2016). The structure of the isolated QUE was confirmed by several technical methods. TLC revealed the same brown spot for the isolated QUE and the standard with R_f_ value of 0.52 after the plate was sprayed with iodine and 5% FeCl_3_. This result was similar to that of Singh et al. (2012) in which the R_f_ value of QUE was confirmed. Moreover, the UV–vis spectroscopy initially confirmed the isolation of quercetin from Fenugreek extract with peaks at 386, 296 and 262 nm compared with standard quercetin. Also, the isolated quercetin was analyzed by FTIR and revealed the presence of a C–O–C bond at 1009 cm^−1^, C=C bond at 1610 cm^−1^, C=O bond at 1667 cm^−1^ and an O–H bond at 3406 cm^−1^ compared with the standard quercetin. The results of UV–visible and FTIR of isolated QUE confirmed that its structure was similar to that previously described (Deore et al. 2013; Tiwari et al. 2015). Therefore, the characterization measurements elucidated the effective isolation of quercetin from Fenugreek extract with a high degree of purity.

To compare the in-silico theoretical study with practical data, the ADA enzyme was isolated from rat joints and assayed in the absence and presence of Fenugreek QUE. The ADA activity was inhibited in rat joints by Fenugreek QUE with an IC_50_ value of 0.17 mM, whereas previous study reported that the IC_50_ value of Reynoutria japonica which represented a natural inhibitor of ADA was 0.629 mM (Zhang et al. 2019). The fenugreek QUE acts as a non-competitive inhibitor with a K_i_ value of 55.5 mM. Moreover, the K_i_ result is novel because there is no evidence in the literatures to compare the results with however, some studies are not consistent with our results (Singh and Sharma 2000; Adamek et al. 2020; Kumar and Sharanya 2020; Kutryb-Zajac et al. 2020). Thus, our results indicated that Egyptian Fenugreek QUE may act as a natural inhibitor of ADA which is a key inflammatory enzyme. This was confirmed theoretically in-silico and in practical studies.

The results showed that combining QUE and MTX therapy had a greater effect on reducing inflammatory symptoms compared with either MTX or QUE treatment alone. The progression of arthritis led to dramatic decrease in rat body weight and an increase in the PV, this was consistent with previous studies reported RA in experimental animals (Rasool et al. 2006; Egan et al. 2004; Granado et al. 2005; Roy et al. 2017). When arthritic rats were compared with their normal control counterparts, they showed signs of arthritis. In contrast to their arthritic control counterparts, QUE-treated arthritic rats reported a marked increase in body weight and decrease in PV. These results are consistent with that of other studies (Rasool et al. 2006; Roy et al. 2017; Kumar et al. 2009; Haleagrahara et al. 2017).

The anti-inflammatory efficacy of QUE alone or in combination with MTX was further confirmed by live imaging and X-ray examination of the animals. RA control rats showed mild to moderate cartilage damage and, bone erosion indicating bone destruction. This may result from the chronic exposure of ankle joints to proinflammatory mediators such as TNF-α and IL-1^β^, which stimulate the production of proteolytic enzymes which result in the degradation of cartilage (Roy et al. 2017). We observed that small joints such as the tarsal, metatarsal, and interphalangeal were more affected in RA rat control group. However, in case of QUE-treated and MTX alone or in the combinational groups, these abnormalities were ameliorated, and in the QUE/MTX combined group, it is bearing more resemblance to radiographic pattern of the joints of normal group. This indicates the enhanced anti-inflammatory efficacy of QUE and MTX.

Local injection of CFA resulted in an increase of TNF-α, IL-1^β^, and IL-6 levels due to activation of autoimmunity cells which aggravated complete inflammation. In contrast, treatment with QUE and/or MTX, showed a reduction in serum TNF-, IL-1^β^, and IL-6 with a minimal reduction in the combined treated group compared with the RA untreated group. Thus, the combination treatment of QUE and MTX was more effective at limiting inflammatory cell infiltration and halting or delaying bone loss. This is largely consistent with the presence of the different pathological patterns observed previously (Haleagrahara et al. 2017).

The immunological markers C-reactive protein (CRP), anti-cyclic citrullinated peptide (anti-CCP) or rheumatoid factor (RF) and ADA enzyme are released when cartilage and bone become inflamed. The presence of these immunological markers in the blood is associated with the onset and progression of arthritis. They are thought to play a part in the progression of RA (Nagatomo et al. 2010; Shadick et al. 2006). Contrary to what was mentioned previously (Shen et al. 2015), our results showed that after 28 days, QUE and MTX therapy significantly decreased CRP, anti-CCP, RF and ADA enzyme with the lowest value for the combined treatment.

Recently, Sangeetha et al. reported a link between RA and oxidative stress. They demonstrated that elevated levels of ROS and oxidative stress caused liver, brain, and cartilage damage in CFA rats (Sangeetha et al. 2020). The data from the present study revealed that arthritic rats and MTX-administrated rats exhibited an impairment in oxidant/antioxidant homeostasis, resulting in a marked increase in MDA levels and degradation of the antioxidant enzyme GPx in the joint supernatant. This finding is consistent with that of a previously study (Elmansy et al. 2021). Furthermore, the combined therapy of QUE and MTX reduced MTX-induced oxidative damage and increased antioxidant enzyme protection in joint tissue as QUE could reduce the production of free radicals. This is in accordance with previous report by Eshwarappa et al. who found that QUE had a hepatoprotective activity against hexachlorocyclohexane and thioacetamide-induced oxidative injury (Eshwarappa et al. 2015).

Previous studies have suggested correlation between ADA activity and RA joint inflammation (Gao et al. 2020; Nair et al. 2020; Gangadharan et al. 2021). The results demonstrated that there was significant increase in ankle joint ADA activity and mRNA levels in RA rats compared with control rats. After 28 days, QUE and MTX significantly decreased the activity and expression levels of ADA enzyme compared with the untreated controls. This may be due to the anti-inflammatory, immunomodulatory, antioxidant, and the regulatory effects of QUE on cell signaling (Almatroodi et al. 2021; Zaragozá et al. 2020; Marefati et al. 2021). Thus, QUE represents a targeted treatment for RA due to its effect on inflammatory cells and molecules and this effect results from its role in inhibition of the ADA inflammatory enzyme.

The histopathology of the arthritic rat ankle joint revealed marked inflammatory conditions. QUE and MTX alone treated, or the combination groups showed a decrease in the sub—periosteum region, destruction of cartilage, synovial membrane and vascular proliferation with reduced levels of chondrocytes and synovial joint space. These observations were seen to a greater extent in the combination drug-treated group compared with the alone treated group indicating a synergistic effect of the combination. Furthermore, X-ray analysis and histopathological assessment also provided evidence for the proper induction of RA in the rats following adjuvant injection and the role of QUE and/or MTX as effective therapy for RA inflammation. This effect resulted from their role in inhibition of ADA inflammation enzyme. These results was in accordance with that of previous studies (Roy et al. 2017; Ebrahimzadeh et al. 2008).

## Conclusion

**Fig. 11:**
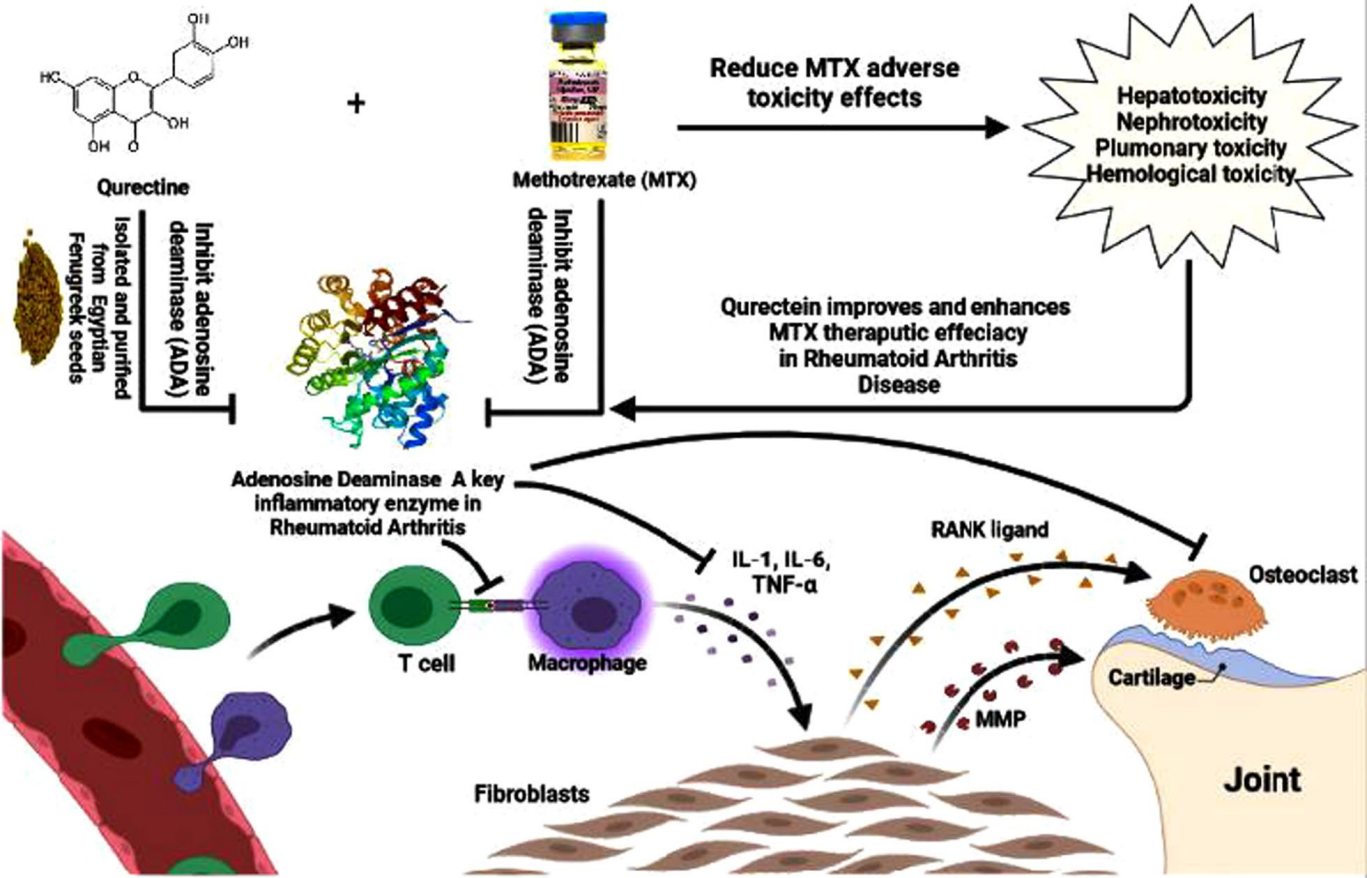
Systematic diagram showed the effect of Egyptian fenugreek quercetin either alone or in combination with MTX in rheumatoid arthritis disease

Collectively, the isolated quercetin from Egyptian Fenugreek seeds exhibits a significant inhibitory effect on adenosine deaminase enzyme (ADA), and inflammatory cytokines biomarkers either alone or in combination with methotrexate (MTX) in a RA rat model. Moreover, Fenugreek quercetin reduced MTX—induced toxicity. Thus, we recommended Egyptian Fenugreek quercetin as a natural adjuvant in mitigating (RA) because of its antiarthritic properties and its ability to improve the therapeutic efficacy of MTX (Fig. 11).

## Data Availability

The datasets used and/or analyzed during the current study are available from the corresponding author upon reasonable request.

## References

[CR1] Abu-Khudir R, Salem MM, Allam NG, Ali EM (2019). Production, partial purification, and biochemical characterization of a thermotolerant alkaline metallo-protease from *Staphylococcus sciuri*. Appl Biochem Biotechnol.

[CR2] Adamek RN, Ludford P, Duggan SM, Tor Y, Cohen SM (2020). Identification of adenosine deaminase inhibitors by metal-binding pharmacophore screening. ChemMedChem.

[CR3] Almatroodi SA (2021). Potential therapeutic targets of quercetin, a plant flavonol, and its role in the therapy of various types of cancer through the modulation of various cell signaling pathways. Molecules.

[CR4] Anderson EW, Fornell C, Lehmann DR (1994). Customer satisfaction, market share, and profitability: findings from Sweden. J Mark.

[CR5] Bedoui Y (2019). Methotrexate an old drug with new tricks. Int J Mol Sci.

[CR6] Bradford MM (1976). A rapid and sensitive method for the quantitation of microgram quantities of protein utilizing the principle of protein-dye binding. Anal Biochem.

[CR7] Cristalli G (2001). Adenosine deaminase: functional implications and different classes of inhibitors. Med Res Rev.

[CR8] Cronstein BN, Aune TM (2020). Methotrexate and its mechanisms of action in inflammatory arthritis. Nat Rev Rheumatol.

[CR9] Deore S, Nikole K, Baviskar B, Khadabadi S (2013). Isolation and quantitative estimation of quercetin in *Lagenaria siceraria* fruit. J Chromatogr Sep Tech.

[CR10] Dua A, Vats S, Singh V, Mahajan R (2013). Protection of biomolecules against in vitro oxidative damage by the antioxidants from methanolic extract of *Trigonella foenum-graecum* seeds. Int J Pharm Sci Res.

[CR11] Ebrahimzadeh MA, Pourmorad F, Bekhradnia AR (2008). Iron chelating activity, phenol and flavonoid content of some medicinal plants from Iran. Afr J Biotechnol.

[CR12] Egan TM, Yang B, Bartlett KR (2004). The effects of organizational learning culture and job satisfaction on motivation to transfer learning and turnover intention. Hum Resour Dev Q.

[CR13] Elmansy RA, Seleem HS, Mahmoud AR, Hassanein EH, Ali FE (2021). Rebamipide potentially mitigates methotrexate-induced nephrotoxicity via inhibition of oxidative stress and inflammation: a molecular and histochemical study. Anat Rec.

[CR14] Eshwarappa RSB, Iyer S, Subaramaihha SR, Richard SA, Dhananjaya BL (2015). Antioxidant activities of *Ficus glomerata* (moraceae) leaf gall extracts. Pharmacogn Res.

[CR15] Firdous S (2014). Phytochemicals for treatment of diabetes. EXCLI J.

[CR16] Gangadharan H, Singh A, Majumder S, Aggarwal A (2021). Adenosine deaminase gene polymorphism and baseline serum level of adenosine deaminase as a biomarker of response to methotrexate in rheumatoid arthritis. JCR.

[CR17] Gao Z-w (2020). The roles of adenosine deaminase in autoimmune diseases. Autoimmun Rev.

[CR18] Gardi C (2015). Quercetin reduced inflammation and increased antioxidant defense in rat adjuvant arthritis. Arch Biochem Biophys.

[CR19] Granado M, Priego T, Martín AI, Villanúa MÁ, López-Calderón A (2005). Anti-inflammatory effect of the ghrelin agonist growth hormone-releasing peptide-2 (GHRP-2) in arthritic rats. Am J Physiol Endocrinol Metab.

[CR20] Guisti G, Bergmeyes HU (1974). Adenosindeaminasa. Methods of enzymatic analysis.

[CR21] Haleagrahara N, Radhakrishnan A, Lee N, Kumar P (2009). Flavonoid quercetin protects against swimming stress-induced changes in oxidative biomarkers in the hypothalamus of rats. Eur J Pharmacol.

[CR22] Haleagrahara N (2017). Therapeutic effect of quercetin in collagen-induced arthritis. Biomed Pharmacother.

[CR23] Haleagrahara N (2018). Flavonoid quercetin–methotrexate combination inhibits inflammatory mediators and matrix metalloproteinase expression, providing protection to joints in collagen-induced arthritis. Inflammopharmacology.

[CR24] Halgren TA, Nachbar RB (1996). Merck molecular force field. IV. Conformational energies and geometries for MMFF94. J Comput Chem.

[CR25] Harder E (2016). OPLS3: a force field providing broad coverage of drug-like small molecules and proteins. J Chem Theory Comput.

[CR26] Kim H-R (2019). Quercetin, a plant polyphenol, has potential for the prevention of bone destruction in rheumatoid arthritis. J Med Food.

[CR27] Kumar GA, Sharanya CS (2020). In silico and in vitro validation of some benzimidazole derivatives as adenosine deaminase inhibitors. Indian J Chem Sect B.

[CR28] Kumar N, Singh S, Patro N, Patro I (2009). Evaluation of protective efficacy of *Spirulina platensis* against collagen-induced arthritis in rats. Inflammopharmacology.

[CR29] Kutryb-Zajac B, Mierzejewska P, Slominska EM, Smolenski RT (2020). Therapeutic perspectives of adenosine deaminase inhibition in cardiovascular diseases. Molecules.

[CR30] Kvastad L (2015). Single cell analysis of cancer cells using an improved RT-MLPA method has potential for cancer diagnosis and monitoring. Sci Rep.

[CR31] Livak KJ, Schmittgen TD (2001). Analysis of relative gene expression data using real-time quantitative PCR and the 2−ΔΔCT method. Methods.

[CR32] Mahmoud MF, Hassan NA, El Bassossy HM, Fahmy A (2013). Quercetin protects against diabetes-induced exaggerated vasoconstriction in rats: effect on low grade inflammation. PLoS ONE.

[CR33] Marefati N (2021). A review of anti-inflammatory, antioxidant, and immunomodulatory effects of *Allium cepa* and its main constituents. Pharm Biol.

[CR34] McInnes IB, Schett G (2017). Pathogenetic insights from the treatment of rheumatoid arthritis. Lancet.

[CR35] Mohamed TM (2006). Adenosine deaminase from camel tick *Hyalomma dromedarii*: purification and characterization. Exp Appl Acarol.

[CR36] Nagatomo F (2010). Effects of exposure to hyperbaric oxygen on oxidative stress in rats with type II collagen-induced arthritis. Clin Exp Med.

[CR37] Nair HG, Singh A, Majumder S, Aggarwal A (2020). EP28 adenosine deaminase-genetic polymorphism and baseline serum level as a biomarker of treatment response to methotrexate in rheumatoid arthritis. Rheumatology.

[CR38] Nalesnik M, Nikolić JM, Jandrić S (2011). Adenosine deaminase and C-reactive protein in diagnosing and monitoring of rheumatoid arthritis. Med Glas.

[CR39] Noser AA, Abdelmonsef AH, El-Naggar M, Salem MM (2021). New amino acid Schiff bases as anticancer agents via potential mitochondrial complex I-associated hexokinase inhibition and targeting AMP-protein kinases/mTOR signaling pathway. Molecules.

[CR40] Prossnitz ER, Barton M (2014). Estrogen biology: new insights into GPER function and clinical opportunities. Mol Cell Endocrinol.

[CR41] Rasool M, Sabina EP, Lavanya B (2006). Anti-inflammatory effect of *Spirulina fusiformis* on adjuvant-induced arthritis in mice. Biol Pharm Bull.

[CR42] Roy T (2017). Effects of co-treatment with pioglitazone and methotrexate on experimentally induced rheumatoid arthritis in Wistar albino rats. Indian J Pharmacol.

[CR43] Salem MM (2020). Propolis potentiates methotrexate anticancer mechanism and reduces its toxic effects. Nutr Cancer.

[CR44] Sambandam B (2016). Extraction and isolation of flavonoid quercetin from the leaves of *Trigonella foenum-graecum* and their anti-oxidant activity. Int J Pharm Pharm Sci.

[CR45] Sangeetha M, Chamundeeswari D, Babu CS, Rose C, Gopal V (2020). Attenuation of oxidative stress in arthritic rats by ethanolic extract of *Albizia procera* benth bark through modulation of the expression of inflammatory cytokines. J Ethnopharmacol.

[CR46] Scott D, Kingsley G (2006). Tumor necrosis factor inhibitors for rheumatoid arthritis. N Engl J Med.

[CR47] Shadick NA (2006). C-reactive protein in the prediction of rheumatoid arthritis in women. Arch Intern Med.

[CR48] Shen R (2015). Rheumatoid factor, anti-cyclic citrullinated peptide antibody, C-reactive protein, and erythrocyte sedimentation rate for the clinical diagnosis of rheumatoid arthritis. Lab Med.

[CR49] Sindhu G, Ratheesh M, Shyni G, Nambisan B, Helen A (2012). Anti-inflammatory and antioxidative effects of mucilage of *Trigonella foenum graecum* (Fenugreek) on adjuvant induced arthritic rats. Int Immunopharmacol.

[CR50] Singh KL, Singh D, Singh VK (2012). Characterization of the molluscicidal activity of *Bauhinia variegata* and *Mimusops elengi* plant extracts against the fasciola vector *Lymnaea acuminata*. Rev Inst Med Trop São Paulo.

[CR51] Singh L, Sharma R (2000). Purification and characterization of intestinal adenosine deaminase from mice. Mol Cell Biochem.

[CR52] Tiwari N, Mishra A, Bhatt G, Chaudhary A (2015). Anti-stress activity of a bioflavanoid: quercetin from *Euphorbia hirta*. J Pharm Res Int.

[CR53] Williams RJ, Spencer JP, Rice-Evans C (2004). Flavonoids: antioxidants or signalling molecules?. Free Radic Biol Med.

[CR54] Zamani B, Jamali R, Jamali A (2012). Serum adenosine deaminase may predict disease activity in rheumatoid arthritis. Rheumatol Int.

[CR55] Zaragozá C, Villaescusa L, Monserrat J, Zaragozá F, Álvarez-Mon M (2020). Potential therapeutic anti-inflammatory and immunomodulatory effects of dihydroflavones, flavones, and flavonols. Molecules.

[CR56] Zhang X-G (2019). *Reynoutria japonica* from traditional Chinese medicine: a source of competitive adenosine deaminase inhibitors for anticancer. Comb Chem High Throughput Screen.

[CR57] Zuo G, Li Q, Hao B (2014). On K-peptide length in composition vector phylogeny of prokaryotes. Comput Biol Chem.

